# A pancreaticobiliary fistula due to pancreatic stones in long-term follow-up of chronic pancreatitis

**DOI:** 10.1055/a-2573-7540

**Published:** 2025-04-15

**Authors:** Kazuya Sumi, Jun Ushio, Takahiro Miyake, Hisaki Kato, Yuki Kawasaki, Takayoshi Ito, Haruhiro Inoue

**Affiliations:** 1 378609 Digestive Diseases Center, Showa University Koto-Toyosu Hospital, Tokyo, Japan

Pancreaticobiliary fistulas (PBFs) are rare and associated with pseudocysts or intraductal papillary mucinous neoplasms 1 2 3 . We report the case of a patient suffering from a PBF caused by pancreatic stones (PSs) that developed during the long-term course of chronic pancreatitis (CP). The stones migrated into the bile duct (BD). The patient was treated by fragmenting the PSs by cholangioscopy, followed by covered self-expanding metal stent (CSEMS) and pancreatic duct (PD) stent placement to facilitate PBF closure.

A man aged 40 years with chronic alcoholic pancreatitis, PSs, and PD stenosis ( Fig. 1 **a–c** ) underwent repeated endoscopic PD dilations and stent placements to manage complications ( Fig. 2 ).

**Fig. 1:**
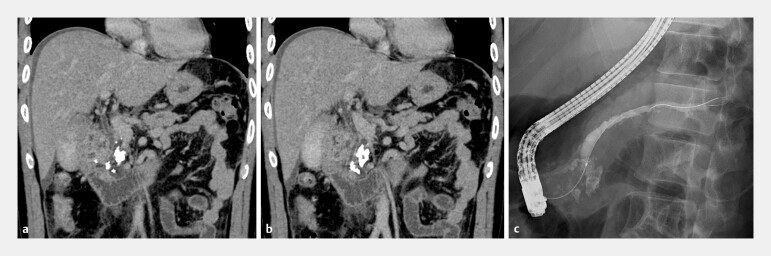
**a, b** Computed tomography images obtained at the initial visit. PSs can be observed in the head of the pancreas. No evidence of BD stenosis is visible. **c** An image from the first ERCP. The main PD in the head is narrowed due to PSs, with dilation of the distal PD. BD, bile duct; ERCP, endoscopic retrograde cholangiopancreatography; PD, pancreatic duct; PS, pancreatic stone.

**Fig. 2:**
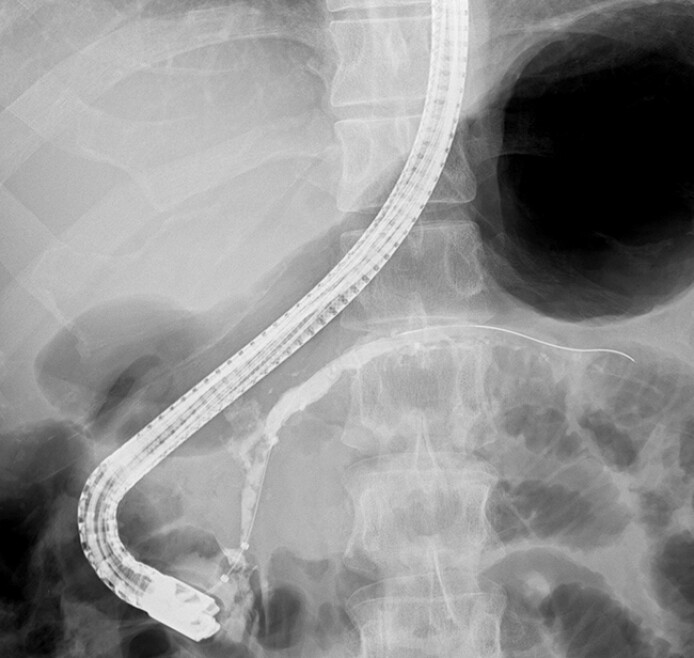
Improvement in the stenosis of the PD in the head and dilation of the distal PD after 32 months of initial ERCP facilitated the extraction of the PD stent. PD, pancreatic duct.

After 1 year of PD stent removal, he presented with fever. Computed tomography revealed liver abscess, and percutaneous drainage was performed. Biliary compression due to PSs, infection, abscess, and biloma were suspected, prompting endoscopic retrograde cholangiopancreatography (ERCP).

Biliary cannulation was successfully performed during ERCP, and a guidewire was advanced through the PBF. Endoscopic cholangiography was performed with contrast to visualize the PD via the PBF, which revealed distal BD stenosis. Biliary and PD stents were placed and cholangioscopy was scheduled ( Fig. 3 **a–c** ).

**Fig. 3:**
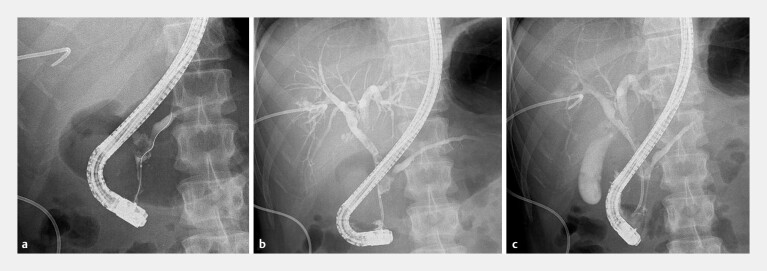
**a** BD cannulation and the guidewire provided easy access to the PD through the PBF. Cholangiography enabled the visualization of the PD at the stone boundary. **b** Visualization of the proximal BD on cholangiography. **c** Plastic stents were inserted into the BD and PD, with a plan for subsequent cholangioscopy. BD, bile duct; PBF, pancreaticobiliary fistula; PD, pancreatic duct.

Follow-up cholangioscopy performed after 2 months revealed biliary stenosis caused by a large white PS. Direct cholangioscopy-guided electrohydraulic lithotripsy, which was performed for fragmenting and extracting the PS, revealed a PBF within the BD. Subsequently, a CSEMS was placed in the BD with the simultaneous plastic stent placement in the PD ( Fig. 4 **a–c** ). However, an ERCP performed 4 months later indicated PBF persistence ( Fig. 5 ; Video 1 ).

**Fig. 4:**
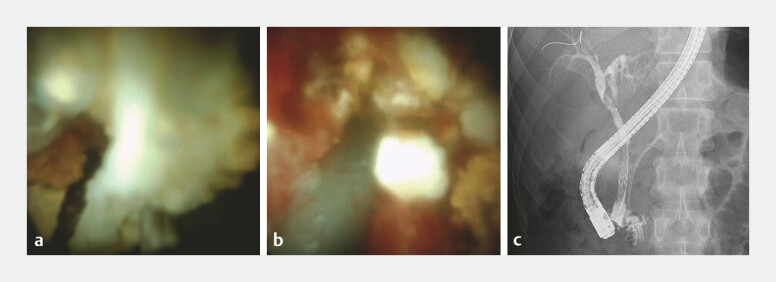
**a, b** Identification of the white PS following the insertion of the cholangioscope into the BD. Visualization of the retained PD stent in the BD following lithotripsy performed with electrohydraulic lithotripsy. **c** A covered self-expanding metal stent was inserted into the BD, and a plastic stent was placed in the PD. Post-insertion cholangiography indicated no evidence of the fistula. BD, bile duct; PD, pancreatic duct; PS, pancreatic stone.

**Fig. 5:**
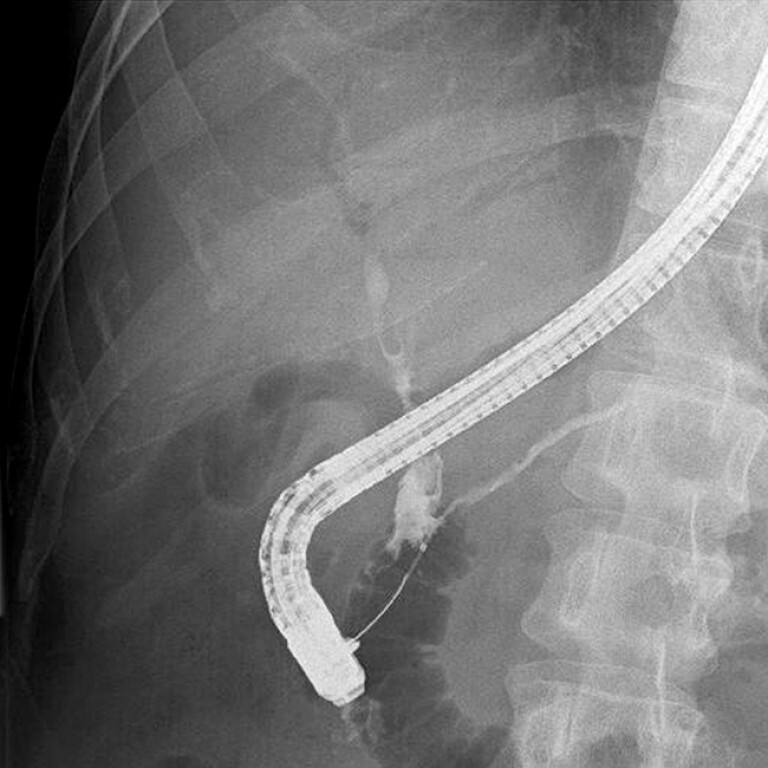
Pancreatography revealed the simultaneous visualization of the bile duct through the fistula. The PBF had not closed. PBF, pancreaticobiliary fistula.

Pancreatic stones, resulting from chronic pancreatitis, developed a pancreaticobiliary fistula over a long period and infiltrated the bile duct.Video 1

To the best of our knowledge, PSs have not been reported to directly cause PBF formation in CP. Given the large fistula, spontaneous closure was not achieved, and surgical treatment was considered. This status is indicative of pancreaticobiliary ductal malfunction and warrants appropriate follow-up.

Endoscopy_UCTN_Code_TTT_1AR_2AI

## Notes

Endoscopy E-Videos https://eref.thieme.de/e-videos
*E-Videos* is an open access online section of the journal *Endoscopy* , reporting on interesting cases and new techniques in gastroenterological endoscopy. All papers include a high-quality video and are published with a Creative Commons CC-BY license. Endoscopy E-Videos qualify for HINARI discounts and waivers and eligibility is automatically checked during the submission process. We grant 100% waivers to articles whose corresponding authors are based in Group A countries and 50% waivers to those who are based in Group B countries as classified by Research4Life (see: https://www.research4life.org/access/eligibility/ ). This section has its own submission website at https://mc.manuscriptcentral.com/e-videos .
